# sPmel17 Secreted by Ultraviolet B-Exposed Melanocytes Alters the Intercellular Adhesion of Keratinocytes

**DOI:** 10.1155/2022/1856830

**Published:** 2022-02-10

**Authors:** Shuang-Hai Hu, Shan Jiang, Fang Miao, Tie-Chi Lei

**Affiliations:** Department of Dermatology, Renmin Hospital of Wuhan University, Wuhan 430060, China

## Abstract

Repigmentation of the skin in patients with vitiligo represents an intricate process in which the depigmented epidermis is replenished by functional melanocytes (MCs) that migrate from undamaged hair follicles and/or surrounding areas. We characterized whether MCs release a secreted form of Pmel17 (sPmel17) protein after exposure to UVB, thereby weakening the cell-cell adhesions of keratinocytes (KCs), which provides MCs the opportunity to migrate to areas devoid of MCs. At first, we examined the interactions of sPmel17 and FHL2 (four-and-a-half LIM domain protein 2) in KCs treated with the conditioned media (CM) from MCs exposed to UVB. The results showed that both the protein and mRNA levels of FHL2 were significantly upregulated in KCs treated with sPmel17-enriched CM from UVB-exposed MCs. We also found that there are physical interactions between sPmel17 and FHL2 as analyzed by reciprocal coimmunoprecipitation assays and double immunofluorescence staining. The CM from UVB-exposed MCs signaled KCs to remodel the actin cytoskeleton and reduce E-cadherin expression. However, the CM from UVB-exposed and Pmel17-silenced or from UVB-unexposed MCs failed to do this. To further determine the in situ distributions of sPmel17, FHL2, and E-cadherin, we examined the expression profiles of those proteins in the skin from healthy subjects and from depigmented or repigmented vitiligo using immunofluorescence and immunohistochemical staining. The results showed that the expression of sPmel17 was positively correlated with FHL2 but not to E-cadherin. The colocalization of FHL2 and sPmel17 was also observed in UVB-exposed mouse tail skin. Together, the upregulation of FHL2 in KCs requires stimulation by sPmel17 secreted from MCs and activation of the sPmel17-FHL2-E-cadherin axis offers a potential therapeutic target to expedite the repigmentation process in patients with vitiligo.

## 1. Introduction

Vitiligo is a chronic, acquired autoimmune disorder characterized by circumscribed depigmented macules in the skin that result from the loss of pigment-producing cells known as melanocytes (MCs) [1, 2]. Although vitiligo is not life-threatening, it can be a social stigma that results in decreased self-esteem among those with that skin condition, and thus, it is inappropriate to simply categorize vitiligo as a cosmetic problem [3]. Vitiligo affects approximately 0.5-2% of the general population worldwide [1]. Phototherapies using narrowband ultraviolet B (NB-UVB), a 308 nm excimer laser, or monochromatic excimer light have been clinically proven to be effective in the treatment of vitiligo [4]. However, the responsiveness to such phototherapies varies greatly in different patients with vitiligo. A recent study indicated that almost 25% of vitiligo patients have a poor response to NB-UVB phototherapy even after 12 months of treatment [5]. It is also a challenge to predict when and where repigmentation will occur in the UVB-treated lesional skin. Light therapy often requires a long duration of treatment over several months, which significantly reduces patient compliance and increases medical costs [5]. The unsatisfactory benefit of current phototherapy regimens prompted us to examine the mechanism behind UVB-induced repigmentation in an attempt to identify new therapeutic targets to improve the treatment outcome for vitiligo patients.

Skin repigmentation represents an intricate process in which the depigmented epidermis is replenished by functional MCs that migrate from undamaged hair follicles and/or from surrounding skin. Cellular events, for example, MCs or melanoblasts that undergo activation, proliferation, migration, and repositioning, seem to be of central importance in the induction of vitiligo repigmentation by UVB therapy [4, 6]. A plethora of KC-derived cytokines, soluble peptides, and proteins, such as alpha-melanocyte stimulating hormone (*α*-MSH) [7] and endothelin-1 [8], has been reported to directly or indirectly stimulate MC proliferation and dendrite elongation; however, very few studies have addressed the role of melanocyte-derived factor(s) in the repigmentation process. Emerging studies have indicated that the melanosomal structural protein Pmel17 is potentially trafficked to the cell plasma membrane and then sheds its ectodomain at the juxtamembrane motif, therefore releasing a soluble secreted form of Pmel17 (termed sPmel17) into the extracellular milieu [9–11]. In this study, we provide evidence for the first time that sPmel17 upregulates the expression of four and a half LIM domains 2 (FHL2), a multifunctional scaffolding protein [12], in KCs. That in turn decreases E-cadherin- (E-cad-) mediated cell-cell adhesion through sPmel17-FHL2 interactions and stimulates actin cytoskeleton remodeling in KCs that creates a microenvironment conducive to melanocyte migration.

## 2. Materials and Methods

### 2.1. Patient Recruitment

Seven well-diagnosed cases (4 males, 3 females) of vitiligo vulgaris were recruited from the Outpatient Department, Renmin Hospital of Wuhan University (RHWU, Wuhan, China). Written informed consent was obtained from each patient, and this study was approved by the Ethics Committee of RHWU. Details are listed in Supplementary Table 2.

### 2.2. Animals and UVB Irradiation

Dct-LacZ transgenic mice were generously provided by Dr. Ian J. Jackson (Western General Hospital, Edinburgh, UK). All mice were kept in specific pathogen-free facilities under a rigorous monitoring system. This study was approved by the Institutional Animal Care and Use Committee of RHWU. The shaved dorsal skin of 8-week-old mice was exposed three times per week for 3 weeks to 500 mJ/cm^2^ UVB from a bank of nine Philips UVB lamps (290-320 nm) with a peak emission of 312 nm (SS-01, Sigma High-Tech Co., Ltd., Shanghai, China). The dose of UVB irradiation was calibrated with a digital radiometer (Sigma High-Tech Co.) before each exposure [13, 14]. The dorsal or tail skin was biopsied from UVB-exposed or UVB-unexposed sites after euthanasia and was then embedded in Tissue-Tek OCT compound (Sakura Finetechnical Co., Ltd., Tokyo, Japan) for subsequential cryosectioning.

### 2.3. Cell Culture, Treatment, and Preparation of Conditioned Medium (CM)

Primary human epidermal MCs and human epidermal KCs were isolated from juvenile foreskin tissues (skin phototype III/IV) as previously described with a minor modification [15]. In brief, foreskin samples were kindly supplied by the Department of Urology, RHWU, and were sterilized with povidone iodine for 10 min and then rinsed with cold phosphate-buffered saline (PBS). Subcutaneous tissue was removed from each biopsy using ophthalmic scissors after which the skin specimens were trimmed into 2 × 4 mm pieces and were then soaked in 0.25% dispase solution (Sigma-Aldrich, St Louis, MO, USA) overnight at 4°C. The epidermis was then separated from the dermis and digested with 0.25% trypsin for an additional 10 min at 37°C to prepare a single-cell suspension. After centrifugation, the cell pellets were resuspended in Medium 254 supplemented with Human MC Growth Supplement to culture primary MCs or in EpiLife medium supplemented with Human KC Growth Supplement (all from Gibco, Invitrogen, Carlsbad, CA, USA) to culture primary KCs. To prepare the CM from MCs, the MCs or transfected MCs were seeded in 6-well plates and were cultured until they reached ~70% confluence. The cells in fresh medium were treated or untreated with 30 mJ/cm^2^ UVB radiation and were then cultured for one additional day. The cell-free supernatants were harvested and further centrifuged and filtered using a 0.22 *μ*m filter to remove cell debris [15, 16]. For treatment of MC-derived CM (MC-CM), the KCs were incubated with MC-CM for 24 h.

### 2.4. Lentivirus-Mediated shRNA for Human Pmel17 Silencing and Quantitative Real-Time RT-PCR (qRT-PCR)

A detailed description of the lentivirus transfection and qRT-PCR protocols is available in the Supplementary Materials. All primer pairs are shown in Supplementary Table S1.

### 2.5. Cell Migration Assay

Cell migration was assessed using Transwell cell culture chambers as previously described [17]. In Transwell chambers (Costar, 3422, Cambridge, MA, USA), polyvinylpyrrolidone-free polycarbonate filters with an 8.0 *μ*m pore size were precoated with 10 mg/mL collagen IV (C6745, Sigma-Aldrich) and then placed on the lower surface of each Transwell chamber and dried overnight at room temperature. The coated filters were washed extensively in PBS and then dried immediately before use. MCs (1 × 10^5^) were seeded into the upper chambers, while the lower chambers were filled with fresh complete Medium 254. MCs were treated with 30 mJ/cm^2^ UVB irradiation. Forty-eight h later, the filters were fixed in methanol and stained with crystal violet. Cells that had migrated into the lower surface of the membranes were counted in five microscopic fields using a 20x objective in at least three independent experiments.

### 2.6. Western Blotting and Coimmunoprecipitation (co-IP) Assay

Cells were harvested, washed in PBS, and lysed in extraction buffer containing 1% Nonidet P-40, 0.01% SDS, and a protease inhibitor cocktail (Roche, Indianapolis, IN, USA). Protein contents were determined using a BCA assay kit (KeyGEN Biotech, Nanjing, China). Equal amounts of each protein extract (20 *μ*g per lane) were resolved using 10% sodium dodecyl sulfate (SDS) polyacrylamide gel electrophoresis (SDS-PAGE). Following transblotting onto Immobilon-P membranes (Millipore, Billerica, MA, USA) and blocking with 5% nonfat milk in saline buffer, the membranes were incubated with the following antibodies, including anti-TYR (ab170905, Abcam, Cambridge, UK, 1 : 1000), anti-MITF (ab3201, Abcam, 1 : 1000), anti-sPmel17 (targeting the N-terminal extracellular domain of Pmel17, sc-377325, Santa Cruz, California, USA, 1 : 1000), anti-Pmel17 (*α*PEP13h, targeting the C-terminus of Pmel17, a gift from Vince Hearing), anti-MCAM (ab75769, Abcam, 1 : 1,000), anti-FHL2 (ab202584, Abcam, 1 : 1,000), anti-DCT (ab74073, Abcam, 1 : 1000), anti-E-cad (ab270257, Abcam), anti-TYRP1 (ab178676, Abcam, 1 : 1000), and anti-GAPDH (ab37168, Abcam, 1 : 10,000) for 1 h at room temperature. The membranes were then washed and incubated with HRP-conjugated anti-rabbit IgG (AS1107, Aspen Biological, Wuhan, China, 1 : 10,000) for 1 h at room temperature. Each membrane was then washed again, and specific immunoreactive bands were visualized using a chemiluminescent reaction (ECL; Amersham, Piscataway, NJ, USA). Signal intensities on the blots were detected using a ChemiDoc Imaging System (Bio-Rad, Hercules, CA, USA), quantified using ImageJ software (NIH, Bethesda, MD, USA), and then normalized to GAPDH. The supernatants were collected and subjected to centrifugation at 5,000 rpm and 4°C for 15 min to remove dead cells and debris. The supernatants were dialyzed overnight against deionized water to remove salts and were then concentrated by lyophilization. To visualize protein bands in the supernatant, the gel was stained with Coomassie blue solution (Beyotime, Nanjing, China). All data were obtained from more than two independent experiments carried out in triplicate.

The interaction of sPmel17 with FHL2 in KCs was evaluated by a reciprocal co-IP assay. KCs treated or untreated with MC-CM were lysed in immunoprecipitation lysis buffer (P0013, Beyotime, Nanjing, China). An anti-sPmel17 or anti-FHL2 primary antibody was added to 200 *μ*L cell lysate. Subsequently, the lysates were incubated overnight at 4°C with gentle rocking, and mouse IgG was used as the negative control. 8 *μ*L protein A plus G agarose beads were added to the mixture and incubated with gentle rocking for 4 h at 4°C, after which the immunoprecipitates were washed 5 times with 500 *μ*L 1× cell lysis buffer. The pellets were resuspended in 20 *μ*L 3× SDS sample buffer, and each sample was heated to 95°C for 5 min, centrifuged for 15 min at 10,000 × g at 4°C, and loaded on SDS-polyacrylamide gels. Western blot analysis was then performed using antibodies against FHL2 and sPmel17.

### 2.7. Immunofluorescence Staining

A detailed description of the immunofluorescence staining protocols is available in the Supplementary Materials. The expression level of immunofluorescence image was quantified by using ImageJ software (NIH, Bethesda, MD), which measures the stained area per microscopic field with consistent threshold.

### 2.8. X-Gal Staining

Tail skins were obtained from Dct-LacZ mice treated or untreated with UVB irradiation. The specimens were fixed in 4% paraformaldehyde at 4°C for 1 h, then embedded in paraffin, and sliced into 5 *μ*m sections for immunohistochemistry. Sections were deparaffinized, rehydrated and microwaved for antigen retrieval, then stained with X-gal staining solution (Beyotime, Nanjing, China) at room temperature for 24 h. LacZ-positive cells were observed using a microscope [18].

### 2.9. Statistical Analysis

Data are expressed as means ± SEM. Student's *t*-test and one-way analysis of variance (ANOVA) were used to compare the mean intensities of western blot bands, migrating cell numbers, and staining intensities. *P* values < 0.05 are considered to be statistically significant. All statistical analyses were performed using GraphPad Prism 6 (San Diego, CA, USA) and SPSS statistical software (version 25, SPSS Inc., Chicago, IL, USA).

## 3. Results

### 3.1. Changes in Phenotypes and the Migration of MCs Exposed to UVB Irradiation

**Figure 1:**
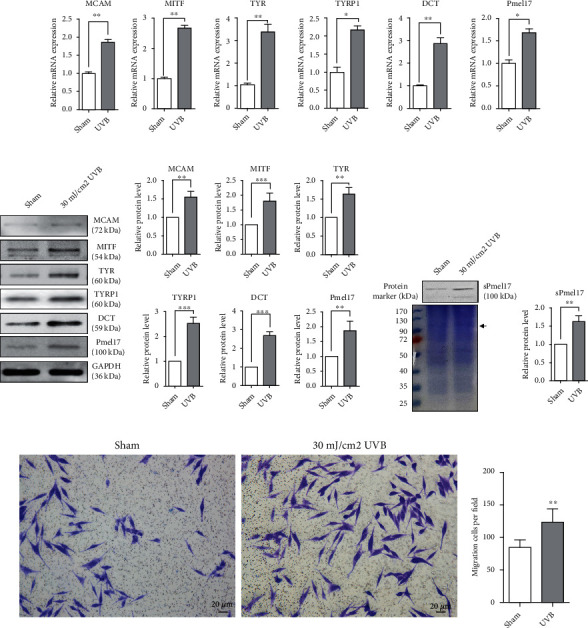
MCs achieve the phenotypes of melanogenesis and migration following UVB exposure. Primary human epidermal MCs were isolated from juvenile foreskin tissues, treated with or without 30 mJ/cm^2^ UVB and then cultured for one additional day. (a) qRT-PCR was performed to measure the expression levels of melanogenesis-associated mRNAs (MITF, TYR, TYRP1, DCT, and Pmel17) and the migration-related MCAM in MCs exposed or unexposed to 30 mJ/cm^2^ UVB. The results were normalized to the housekeeping gene GAPDH. The data represent means ± SD from 3 independent experiments. ^∗^*P* < 0.05; ^∗∗^*P* < 0.01. (b) MCs were lysed in extraction buffer, after which specific antibodies against human MITF, TYR, TYRP1, DCT, Pmel17, and MCAM were used in western blotting to examine their corresponding protein levels. Protein loading variations were determined by immunoblotting with an anti-GAPDH antibody. Representative blots are shown on the left. The histograms show the densitometric quantification of data with means ± SD from 3 independent experiments. ^∗^*P* < 0.05; ^∗∗^*P* < 0.01. (c) Supernatants were collected from UVB-exposed and from unexposed MCs, and the protein concentration in each supernatant was measured using the BCA assay, after which the supernatants were further concentrated by lyophilization as described in Materials and Methods. Levels of sPmel17 in the supernatants from UVB-exposed or UVB-unexposed MCs detected by western blotting are shown (upper panel, right) (^∗∗^*P* < 0.01). An equal amount of total protein (20 *μ*g) of each supernatant was loaded per lane and was separated by SDS-PAGE. A gel stained with Coomassie blue solution is shown (left panel). The black arrowhead indicates the band at 100 kDa of presumed sPmel17. (d) Representative images for cell migration assessed by the Transwell assay (scale bars = 20 *μ*m). Histograms show the number of cells passing through the insert membrane. The data represent means ± SD from 3 independent experiments. ^∗∗^*P* < 0.01.

Although the UVB-induced proliferation and migration of MCs are a well-documented mechanism underlying repigmentation of the skin in patients with vitiligo who undergo phototherapy [6], the precise molecular/cellular process by which MCs travel through the closely connected KCs is still unknown. We examined the mRNA expression levels of melanogenesis-associated genes (MITF, TYR, TYRP1, DCT, and Pmel17) and the migration-related gene MCAM (melanoma cell adhesion molecule) in MCs exposed or unexposed to 30 mJ/cm^2^ UVB. qRT-PCR analysis showed that exposure to UVB significantly upregulates the expression of all melanogenesis-associated genes and MCAM at the transcriptional level (Figure 1(a)). Similar results in protein levels were obtained using western blotting (Figure 1(b)). Interestingly, an increased level of sPmel17 protein was seen in the CM from UVB-exposed MCs (Figure 1(c)). Moreover, Transwell assay results indicated that UVB significantly promotes MC migration on a collagen IV-coated surface (Figure 1(d)). These findings suggest that UVB-exposed MCs achieve an active motile phenotype and release sPmel17 protein into the extracellular environment.

### 3.2. Changes in Cytoskeleton Remodeling and E-cad-Mediated Adhesion of KCs Treated with the CM from UVB-Exposed MCs

**Figure 2:**
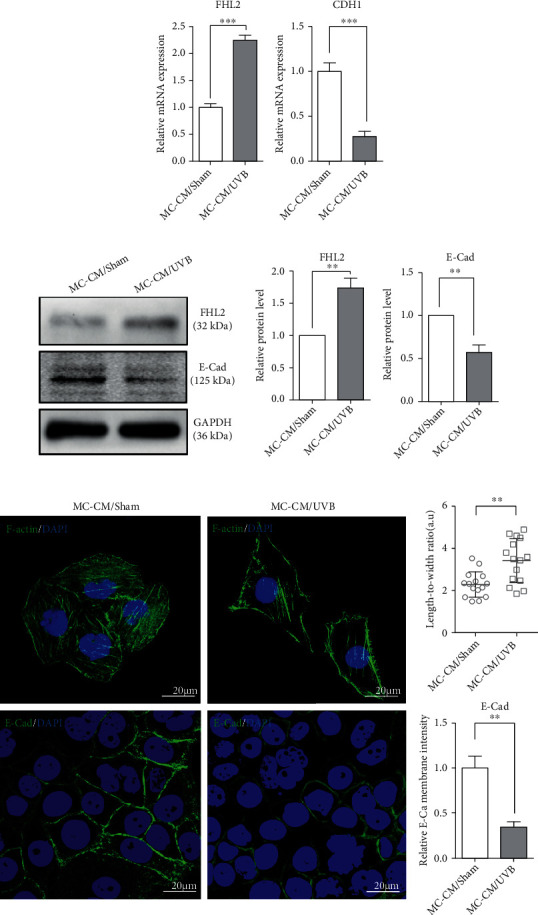
Expression profiles of FHL2 and E-cad in KCs treated with the CM from UVB-exposed or UVB-unexposed MCs. Primary human epidermal KCs were isolated from juvenile foreskin tissues, then treated with the CM from MCs, which had been exposed or unexposed to 30 mJ/cm^2^ UVB. (a) qRT-PCR was performed to measure mRNA levels of FHL2 and CDH1 (encoding E-cad) in KCs treated with the CM from UVB-exposed (MC-CM/UVB) or UVB-unexposed (MC-CM/Sham) MCs. The results were normalized to the housekeeping gene GAPDH. The data represent means ± SD from 3 independent experiments. ^∗^*P* < 0.05; ^∗∗^*P* < 0.01. (b) Protein levels of FHL2 and E-cad were analyzed by western blotting. Representative blots are shown on the left. The histograms show the densitometric quantification of data with means ± SD from 3 independent experiments. ^∗^*P* < 0.05; ^∗∗^*P* < 0.01. (c) Representative images of immunostaining for F-actin (labeled by FITC-phalloidin; upper panel) and E-cad (lower panel). Nuclei were counterstained with DAPI (blue). Histograms (on the right) show the length-to-width ratio and the fluorescence intensity of 20 cells. Data represent means ± SD of three independent experiments. ^∗∗^*P* < 0.01. Scale bars = 20 *μ*m.

Emerging evidence indicates that FHL2 is involved in cytoskeleton remodeling [19] and E-cad-mediated adhesion provides a strong attachment between MCs and KCs and between KCs themselves [20]. Treatment with HMGS culture medium can inadvertently induce the differentiation of KCs, which would impact our analysis. In order to avoid the effects of HMGS on KCs, we used EpiLife medium to culture MCs and to prepare CM after UVB irradiation. To address whether sPmel17 released by MCs modulates the FHL2-driven cytoskeleton remodeling and the E-cad-mediated adhesion of KCs, we first collected the CM from MCs (MC-CM) and then examined the expression levels of FHL2 and E-cad as well as the cytoskeletal F-actin pattern in KCs treated with MC-CM. The mRNA and protein expression levels of FHL2 were significantly increased in KCs treated with the CM from MCs exposed to UVB (MC-CM/UVB) compared with KCs treated with the CM from UVB-unexposed MCs (MC-CM/Sham), whereas decreased amounts of E-Cad at both the mRNA and protein expression levels were seen in KCs treated with MC-CM/UVB (Figures 2(a) and 2(b)). The suppressed expression of E-cad in KCs treated with MC-CM/UVB was also confirmed using immunofluorescence staining (Figure 2(c)). The F-actin staining pattern in KCs treated with MC-CM/Sham appeared as a coarse actin filament pattern throughout the cytoplasm. In contrast, F-actin was distributed in a fine network-like pattern in the cytoplasm of elongated KCs treated with MC-CM/UVB (Figure 2(c)). These results demonstrate that sPmel17 in the MC-CM/UVB may impact the FHL2-driven cytoskeleton remodeling and the E-cad-mediated adhesion of KCs.

### 3.3. A Physical Interaction between sPmel17 and FHL2 Is Required for FHL2-Driven Cytoskeleton Remodeling and E-cad-Mediated Adhesion in KCs

**Figure 3:**
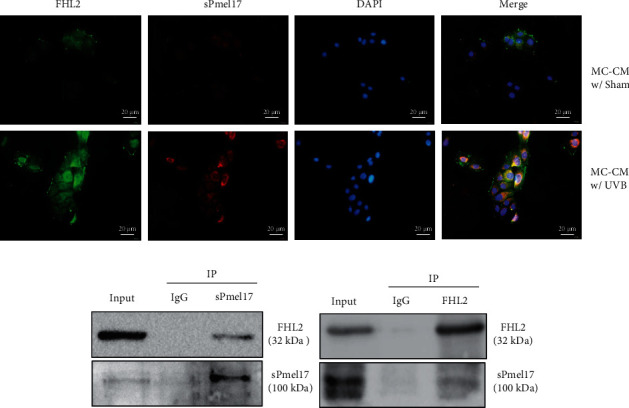
The existence of physical interactions between sPmel17 and FHL2. (a) Representative images of immunofluorescence costaining of FHL2 (green) and sPmel17 (red) in KCs treated with CM from UVB-exposed (MC-CM/UVB, lower panel) or UVB-unexposed MCs (MC-CM/Sham, upper panel). DAPI (blue) was used to visualize cell nuclei. Scale bars = 20 *μ*m. (b) Reciprocal coimmunoprecipitation assay to analyze the interactions between sPmel17 and FHL2. Lysates of KCs treated with MC-CM/UVB were pulled down with an antibody against sPmel17, and the resulting immune complexes were subsequently revealed by western blotting using an antibody against FHL2; representative blots are shown on the left. Anti-FHL2 antibody pulled down sPmel17 (right). Similar results were obtained from 3 independent experiments.

The existence of physical interactions between sPmel17 and FHL2 was examined using coimmunoprecipitation (co-IP) assays and immunofluorescence staining (Figure 3(a)). Lysates of KCs treated with MC-CM/UVB were immunoprecipitated with an antibody against sPmel17 and were then subjected to western blotting analysis using an antibody against FHL2 (Figure 3(b)). There was a clear band corresponding to the 32 kDa band of FHL2 in cellular proteins immunoprecipitated from KC lysates by the antibody against sPmel17. Moreover, a 100 kDa sPmel17 protein was also immunoprecipitated from KC lysates by the antibody against FHL2. As expected, dual immunofluorescence staining showed that sPmel17 and FHL2 colocalized in the cytoplasm of KCs treated with MC-CM/UVB. These data imply that MCs play a role in the regulation of neighboring KCs *in vitro* through sPmel17-FHL2 interactions.

**Figure 4:**
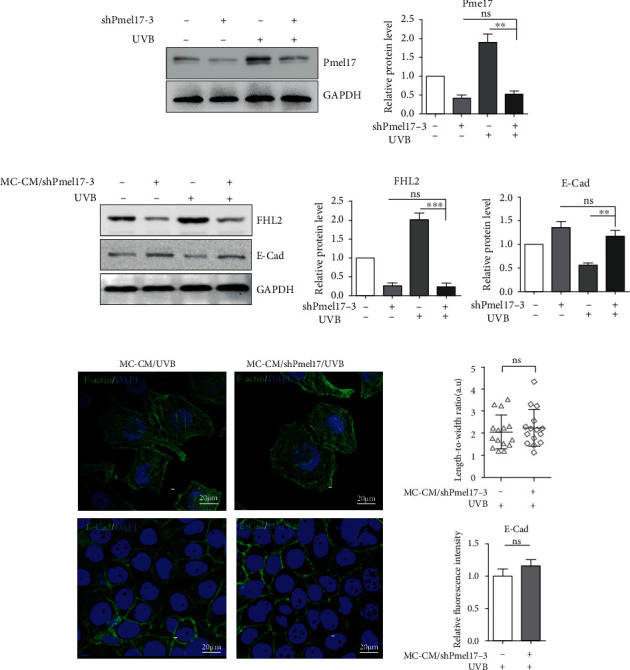
Silencing of Pmel17 in MCs by lentivirus-based shRNA fails to affect the expression of FHL2 and E-cad and the cytoskeletal remodeling of KCs. (a) MCs were transfected with shRNA #3 and then were irradiated with 30 mJ/cm^2^ UVB. Western blotting was performed to confirm the protein levels of Pmel17. The histogram shows the densitometric quantification of data with means ± SD from 3 independent experiments. ^∗^*P* < 0.05; ^∗∗^*P* < 0.01. (b) KCs were treated with CM from MCs transfected with or without Pmel17 shRNA (MC-CM/shPmel17-2) or irradiated with or without 30 mJ/cm^2^ UVB. Western blotting was performed to detect the protein levels of FHL2 and E-cad; representative blots are shown on the left. The histograms show the densitometric quantification of data with means ± SD from 3 independent experiments. ^∗^*P* < 0.05; ^∗∗^*P* < 0.01. (c) Representative images of immunostaining for F-actin (green, upper panels) and E-cad (green, lower panels). DAPI (blue) was used to visualize cell nuclei. Scale bars = 20 *μ*m. Histograms (on the right) show the length-to-width ratio and the fluorescence intensity of 20 cells. Data represent means ± SD of three independent experiments. ns: no significant difference.

To gain more insight about the roles of sPmel17-FHL2 in KCs treated with MC-CM, we designed shRNA vectors targeting the Pmel17 gene in MCs (Figure 4(a), S1) and examined the effects of the CM from MCs transfected with the Pmel17 shRNA lentiviral vector (MC-CM/shPmel17) on the expression levels of FHL2 and E-cad as well as the cytoskeletal F-actin pattern in KCs, using a shRNA scramble plasmid as a negative control. The results showed that CM from MCs transfected with the Pmel17 shRNA but not with the scramble shRNA significantly suppressed the upregulation of FHL2 in KCs (Figure 4(b)). There was a slight increase in E-cad protein levels in KCs treated with the MC-CM/shPmel17 compared to the scramble control, and no change in the F-actin pattern was discerned in KCs treated with the MC-CM/shPmel17 compared to those treated with MC-CM, even after exposure to UVB (Figure 4(c)). Those results lead to the conclusion that sPmel17 released by MCs interacts with FHL2, which modulates cytoskeleton remodeling and E-cad-mediated adhesion in KCs.

### 3.4. In Situ Distribution of sPmel17 and FHL2 in UVB-Exposed Mouse Tail Skin and Repigmented Epidermis of Human Vitiligo

**Figure 5:**
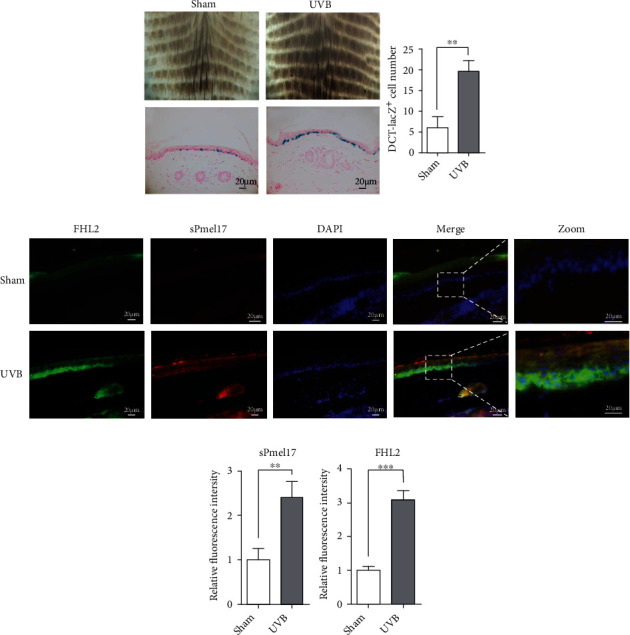
Expression profiles of FHL2 and sPmel17 in UVB-exposed mouse tail skin. (a) The tail skin of Dct-LacZ transgenic mice was irradiated with 500 mJ/cm^2^ UVB three times a week for a total of 9 times over 3 weeks. Representative dermoscope images of UVB-exposed mouse skin are shown in the upper panels. The tail skin was biopsied for cryosectioning and was stained by X-gal solution (lower panels). Histograms (on the right) show the number of LacZ-positive cells in 10 fields. Data represent means ± SD of three independent experiments. ^∗∗^*P* < 0.01. Scale bars = 20 *μ*m. (b) Representative images of immunofluorescence costaining of FHL2 (green) and sPmel17 (red) in UVB-exposed (lower panels) or UVB-unexposed (upper panels) mouse tail skin. Higher magnifications of the boxed areas in the merged images are shown on the far right. (c) Histograms show the fluorescence intensity of 10 fields. Data represent means ± SD of three independent experiments. ^∗∗^*P* < 0.01. Scale bars = 20 *μ*m.

**Figure 6:**
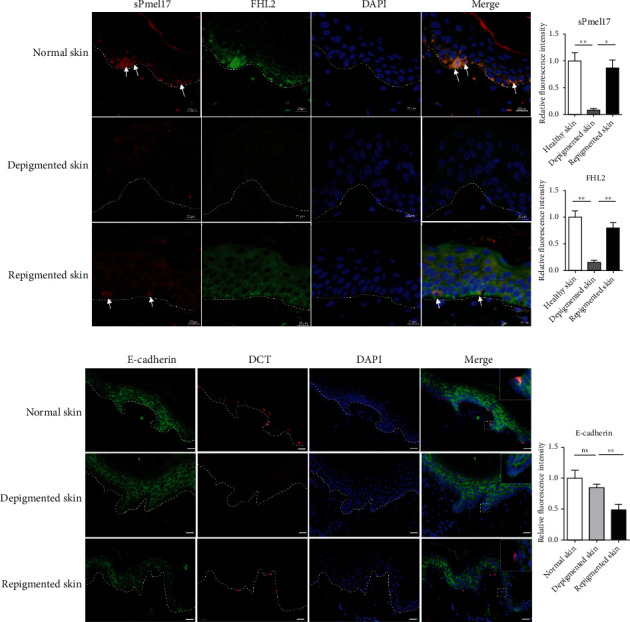
Expression profiles of FHL2/sPmel17 and E-cad/Dct in matched skin from healthy subjects and from depigmented and from repigmented vitiligo. Skin specimens were biopsied from normal skin, depigmented skin, and repigmented skin. (a) Representative images of immunofluorescence costaining of FHL2 (green) and sPmel17 (red) in normal skin (upper panels), in depigmented lesional skin (middle panels), and in repigmented lesional skin (lower panels). Scale bar = 20 *μ*m. (b) Representative images of immunofluorescence costaining of E-cadherin (green) and DCT (red) in biopsies collected from normal skin, depigmented skin, and repigmented skin, high-magnification images of the boxed areas in merged image panel. Scale bar = 50 *μ*m. Histograms show the FHL2, sPmel17, and E-cadherin relative fluorescence intensity of 10 fields. Data represent means ± SD of three independent experiments. ^∗^*P* < 0.05; ^∗∗^*P* < 0.01.

We then examined the expression profile of sPmel17 and FHL2 in the tail skin of Dct-LacZ transgenic mice treated with 500 mJ/cm^2^ UVB, 3 times weekly for 3 weeks. Darkened skin was clearly observed at the UVB-exposed areas, and the numbers of LacZ-positive cells were much higher in UVB-exposed skin than in Sham-exposed skin (Figure 5(a)). Dual immunofluorescence staining (Figure 5(b)) showed that sPmel17 and FHL2 colocalized in the UVB-exposed epidermis with an intensified immunolabeling in the basal and suprabasal layers, which indicates that sPmel17 released by MCs disperses along the basal layer and particularly stimulates basal KCs to express FHL2. Instead, there were no identifiable sPmel17-positive cells in the hair follicles of UVB-exposed or UVB-unexposed mouse tail skin, and only a small number of FHL2-positive cells were discerned in the outer root sheath (ORS), especially in UVB-exposed mouse tail skin (Fig. S2). Furthermore, skin biopsy specimens were taken at the site of vitiliginous lesions and repigmented skin from 7 vitiligo patients with UVB-induced repigmentation and the expression profiles of sPmel17 and FHL2 were detected in those specimens using immunohistochemical staining. The results showed that immunoreactivities with anti-sPmel17 and anti-FHL2 antibodies almost completely disappeared in the vitiliginous lesions; however, the restoration of immunostaining using those same antibodies was found in the repigmented skins from the same vitiligo patients (Figure 6(a)). Finally, to explore the adhesion between MCs and KCs and the epidermal intercellular spaces, expression of the cell–cell adhesion molecule E-cad was analyzed. A positive reactivity for E-cad was detected in all epidermal layers in depigmented skin, whereas an overall decrease in the expression of E-cad was observed in the repigmented skin specimens (Figure 6(b)). These observations support the concept that the abnormal expression of sPmel17 and FHL2 in the epidermis is involved in the pathogenesis of vitiligo and that active interactions of sPmel17 and FHL2 are required for vitiligo repigmentation.

## 4. Discussion

Clinical studies have proven that UVB-based phototherapy can induce perifollicular and marginal repigmentation patterns in the skin of vitiligo patients. It is, however, difficult to conceive how MCs can easily exit from their tightly interconnected epidermal microenvironment to reenter different locations in the skin to establish new networks with neighboring KCs [4, 17, 21]. The coexistence of three types of cells, namely, epidermal MCs and KCs and dermal fibroblasts, in the skin forms a self-regulating homeostatic system, which is highly regulated by an elaborate network of paracrine factors synthesized mainly by epidermal KCs and dermal fibroblasts [22], but so far, very little attention has been paid to MC-derived factors (or proteins) for remodeling of cell-cell adhesions of KCs associated with UVB-induced repigmentation. Several research groups [9–11] independently identified that the melanosomal structural protein Pmel17 is potentially trafficked to the cell plasma membrane and then sheds its ectodomain at the juxtamembrane motif, therefore releasing a soluble secreted form of Pmel17 (termed sPmel17) into the intercellular spaces of the epidermis. However, the roles of sPmel17 in physiological and pathological conditions were unknown until now.

To shed light on the effect of sPmel17 secreted by MCs following UVB radiation on the cell-cell adhesion of KCs, we first evaluated the expression of a panel of specific MC markers including MITF, TYR, TYRP1, DCT, and Pmel17 and the migration marker MCAM in UVB-exposed MCs. As expected, the exposure to UVB caused MCs to achieve the phenotypes of melanogenesis and migration. An increased level of sPmel17 protein was also detected in the CM from UVB-exposed MCs (Figure 1). An augmented production of FHL2 and a decreased expression of E-cad by KCs were found after stimulation with the CM from UVB-exposed MCs but not from UVB-unexposed MCs (Figure 2). On the basis of these observations, we further analyzed the interaction between FHL2 and sPmel17 using reciprocal co-IP assays and dual immunofluorescence staining. The results showed that sPmel17 binds FHL2 to modulate E-cad expression and the F-actin pattern in KCs, findings that were confirmed in independent experiments using a specific shRNA that selectively reduces the secretion of sPmel17 protein (Figures 3 and 4).

FHL2 belongs to the LIM-domain protein family and contains four and a half LIM (LIN-1, ISL-1, and MEC-3) domains, each of which is composed of two zinc finger structures that mediate specific contacts between proteins that participate in the formation of multiprotein complexes [23]. It has been well-documented that FHL2 exerts specific interactions with proteins such as androgen receptor [24], integrin beta subunits [25], and E-cad [26] that have diverse functions. The repigmented skin observed in areas between the grafts was due to the activation of MCs at the donor site. This suggested that continuous enlargement of the intercellular space may be related to the decrease of E-cad expression and that MCs begin to migrate to the lesional skin [27]. In this study, the reciprocal coimmunoprecipitation assay clearly showed that sPmel17 is able to bind FHL2 and further downmodulates E-cad expression in KCs (Figure 3). These findings raise the question of whether the secretion of sPmel17 by UVB-exposed MCs stimulates the expression of FHL2 in KCs, which is negatively correlated to E-cad-mediated cell adhesion. To this end, we used immunofluorescence and immunohistochemistry to examine the expression profiles of FHL2 and sPmel17 in UVB-treated tail skin of Dct-LacZ transgenic mice and the repigmented lesional skins from vitiligo patients who underwent UVB therapy. We found that the distribution of sPmel17 was quite similar to FHL2 in UVB-exposed skin and that the expression of sPmel17 correlated positively to FHL2 but negatively to E-cad (Figures 5 and 6). However, we observed no identifiable sPmel17-positive cells in the hair follicles of UVB-exposed or UVB-unexposed mouse tail skin, and only a small number of FHL2-positive cells were discerned in the outer root sheath (Fig S1). UVB fails to upregulate sPmel17 and FHL2 in mouse hair follicles, so we assume that the activation mechanism of the sPmel17/FHL2/E-cadherin axis by UVB irradiation might play a role in the later stage of perifollicular repigmentation that occurred in patients with vitiligo, in which melanoblasts become mature melanocytes to release excessive amounts of sPmel17.

Taken together, a systematic study focusing on the regulation of intercellular adhesion of KCs by MC-derived factors has, to our knowledge, not been previously performed. Our findings demonstrate that UVB-exposed MCs modulate the E-cad-mediated cell-cell adhesion of KCs through activation of the sPmel17-FHL2-E-cad axis, which creates a microenvironment conducive to MC migration and may serve as an intervention target to expedite the marginal and perifollicular repigmentation process of vitiligo.

## Data Availability

The data that support the findings of this study are available from the corresponding author upon reasonable request.

## Supplementary Materials

Supplementary Materials Materials and Methods. Figure S1: silencing of Pmel17 in MCs by lentivirus-based shRNA. Figure S2: expression profiles of FHL2 and sPmel17 in the hair follicles of UVB-exposed mouse tail skin. Supplementary Table 1: the sequences of primers used in qRT-PCR. Supplementary Table 2: demographic data of patients and healthy subjects.
