# Supplementary material for: sPmel17 Secreted by Ultraviolet B-Exposed Melanocytes Alters the Intercellular Adhesion of Keratinocytes
Source: Oxid Med Cell Longev. 2022 Feb 10;2022:1856830. doi: 10.1155/2022/1856830 (PMC8853777; doi:10.1155/2022/1856830)
Supplement: Supplementary Materials — Materials and Methods. Figure S1: silencing of Pmel17 in MCs by lentivirus-based shRNA. Figure S2: expression profiles of FHL2 and sPmel17 in the hair follicles of UVB-exposed mouse tail skin. Supplementary Table 1: the sequences of primers used in qRT-PCR. Supplementary Table 2: demographic data of patients and healthy subjects. [file 1856830.f1.docx]

**Supplementary Materials**

**sPmel17 secreted by ultraviolet B-exposed melanocytes alters intercellular adhesion of keratinocytes**

*Shuang-Hai Hu1, Shan Jiang1, Fang Miao1, Tie-Chi Lei1#*

*Department of Dermatology, Renmin Hospital of Wuhan University, Wuhan 430060, China*

**Materials and Methods**

***Quantitative real-time RT-PCR (qRT-PCR)***

Total RNAs were extracted using RNA isolator (R401-01, Vazyme, Nanjing, China) according to the instructions of the manufacturer. cDNAs were synthesized from total RNAs using a HiScript II Q Select RT SuperMix (R223, Vazyme). PCR was performed in triplicate with ChamQ Universal SYBR qPCR Master Mix (Q711, Vazyme), 50 ng cDNA and 1 mM forward and reverse primers for the genes of interest. The sequences of primers used in PCR are listed in Table S1. Real-time PCR was performed using an ABI 7500 system with the following cycle parameters: denaturation at 95°C for 30 s, followed by 40 cycles of 95°C for 10 s, 60°C for 30 s and 72°C for 30 s. The purity of each PCR product was checked by dissociation curve analysis as well as by running each sample on 1% agarose gels. Fold change values were calculated using the formula of 2 ^^△△Ct^.

***Lentivirus-mediated shRNA for human Pmel17 silencing***

Lentiviral human Pmel17-targeting short hairpin RNA (shRNA) was purchased from Shanghai GeneChem Co., Ltd. (Cat#: GIEL0241023, Shanghai, China). The shRNAs for RNA interference against human Pmel17 mRNA were designed on the basis of the human Pmel17 cDNA sequence (GenBank accession number NM_001200054) and targeted the regions of nucleotides 1926-1946 (shRNA#1, 5’-CAGACTTATGAAGCAAGACTT-3’), 1379-1399 (shRNA#2, 5’-CAATCATGTCTACGGAAAGTA-3’) and 1037-1057 (shRNA#3, 5’-CTACAGAAGTTGTGGGTACTA-3’). A scrambled shRNA was used as a negative control. The cells were transfected with lentivirus particles following the instructions of the manufacturer, after which clones of cells positively transfected with the recombinant lentivirus were purified by treatment with 3.5 μM puromycin for 1 week.

***Immunofluorescent staining***

Primary KCs were seeded in 6-well culture plates containing coverslips. After attachment, the cells were treated or untreated with MC-CM for 12 h. The cells were then immediately fixed in 4% paraformaldehyde in PBS for 30 min at room temperature, permeabilized with 0.2% Triton X-100 in PBS for 10 min and then blocked for 1 hr at 37°C using a blocking buffer including 10% normal goat serum. The primary antibodies, including anti-FHL2 (ab202584, Abcam, 1:1,000), anti-sPmel17 (sc-377325, Santa Cruz), anti-E-cad (ab270257, Abcam)，anti-DCT (ab74073, Abcam) and anti-F-actin/Phalloidin (A22281, Sigma-Aldrich) were diluted to 1:100 in blocking buffer and were placed on the cells at 4°C overnight. After the incubation, cells on coverslips were washed three times in PBS and were then incubated with goat anti-rabbit IgG (H+L) conjugated with Cy3/FITC secondary antibody (Servicebio, Wuhan, China) for 1 hr at 37°C. For immunofluorescent double-staining, the cryosections of mouse tail skins were incubated with a mixture of rabbit anti-FHL2 and mouse anti-sPmel17 antibodies. Nuclei were stained using 4′6′-diamidino-2-phenylindole (DAPI) solution. Imaging was performed using an FV1200 (Olympus, Tokyo, Japan) confocal microscope.

Figure S1


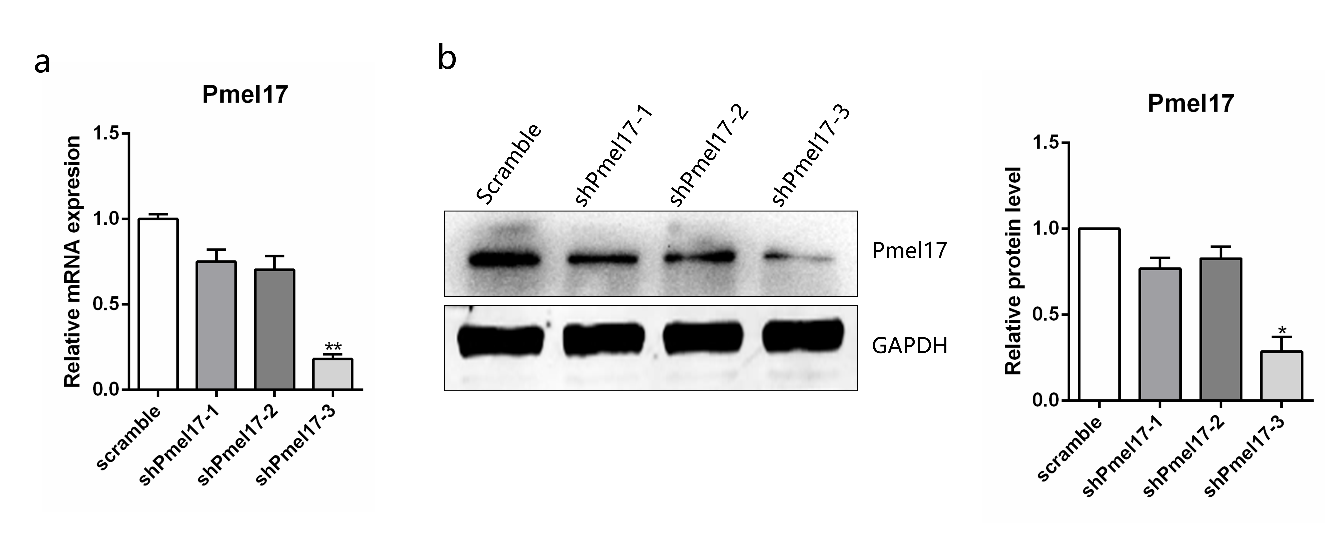


**Figure S1. Silencing of Pmel17 in MCs by lentivirus-based shRNA**. Three different shRNAs (#1，#2 and #3) targeting exons 5 and 10 of the Pmel17 gene transcript were purchased from Shanghai GeneChem Co., Ltd (Cat#: GIEL0241023, Shanghai, China). (a) The MCs were transfected with those three lentivirus-based shRNAs to create stable cell clones for Pmel17 silencing and the mRNA expression levels of Pmel17 in puromycin-resistant cell clones were determined by qPCR. The histogram (on the left) shows the Pmel17 expression level normalized to the GAPDH housekeeping gene, in shRNA-transfected MCs relative to MCs transfected with the scramble control (non-silencing) (100%). (b) Western blotting was performed to measure protein levels in shRNA-transfected MCs. Representative blots are shown in the right. The histogram on the right shows the densitometric quantification of data with means ± SD from 3 independent experiments. **P* <0.05, ***P* <0.01.

Figure S
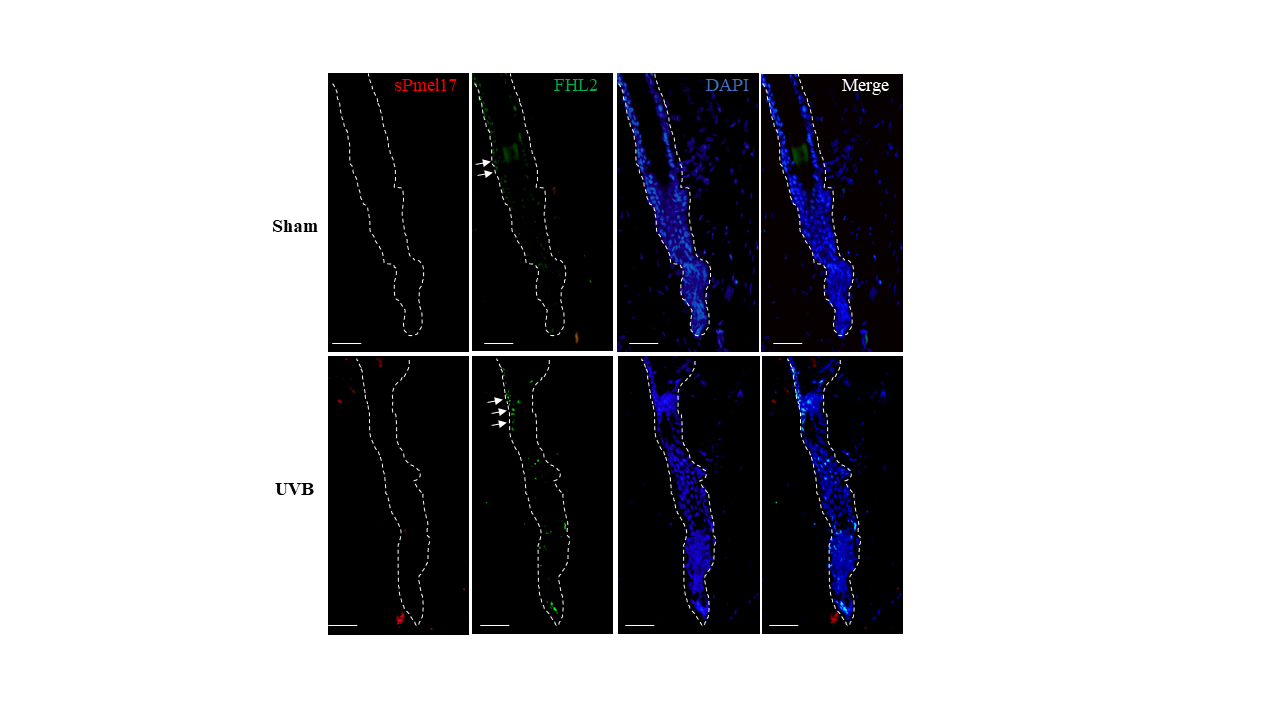
2

**Figure S2. Expression profiles of FHL2 and sPmel17 in the hair follicles of UVB-exposed mouse tail skin.** The longitudinal section of an anagen hair follicle in UVB-exposed (lower panel) or -unexposed (upper panel) mouse tail skin was made by cryosectioning. The expression and distribution of FHL2 and sPmel17 were observed by using dual immunofluorescent staining. Representative images of immunofluorescence co-staining of sPmel17 (red) and FHL2 (green) in mouse hair follicles are shown. White arrows indicate the FHL2-positive cells in the outer root sheath. Scale bar = 20 μm.

**Supplementary Table 1. The sequences of primers used in qRT-PCR**

| **Primer name** | **Species** | **Sequence (5' to 3')** | **Accession number** |
| --- | --- | --- | --- |
| h-GAPDH | Homo | F: CAATGAATACGGCTACAGCA  R: AGGGAGATGCTCAGTGTTGG | XM_034486355 |
| h-MITF | Homo | F: CAAATACGTTGCCTGTCTCGG  R: TGGCCAGTGCTCTTGCTTCA | NM_006722 |
| h-TYR | Homo | F: GCTATCTACAAGATTCAGACCCAGA  R: TGACGACACAGCAAGCTCAC | XM_003832989 |
| h-TRP1 | Homo | F: ACCAGAGGGTTCTCATAGTCAG  R: TGACGACACAGCAAGCTCAC | XM_034987902 |
| h-DCT | Homo | F: CGGTGGACAGCCTAGTGAAC  R: TAGCCGGCAAAGTTTCCTGT | XM_003832021 |
| h-FHL2 | Homo | F: GGCTGTGACTGCAAGGACT  R: GTGGCAGATGAAGCAGGTCT | XM_035001879 |
| h-PMEL | Homo | F: ATTGTCCAGGGTATTGAAAGTGCC  R: CCAGGCATGATAAGCTGGGTG | NM_006928 |
| h-MCAM | Homo | F: CGTCTCGTAAGAGCGAACTTG  R: GTATTTCTCTCCCTGGTCTCCC | NM_006500 |
| h-CDH1 | Homo | F: GGTGCTCTTCCAGGAACCTC  R: CAGCTGTTGCTGTTGTGCTT | XM_034940548 |

**Supplementary Table 2. Demographic data of patients and healthy subjects**

| **Case** | **Type of vitiligo** | **Age/** G**ender** | **Location of skin involved** | **Treatments** | **Onset of response (day)** | **The Outcome and pattern of repigmentation** |
| --- | --- | --- | --- | --- | --- | --- |
| 1 | NSV | 35/Male | Root of neck | Surgical excision | / | 100% |
| 2 | NSV | 20/Female | Left forearm | Surgical excision | / | 100% |
| 3 | NSV | 40/Male | Neck | Surgical excision | / | 100% |
| 4 | NSV | 23/Female | Right forehead | NB-UVB for 10 weeks | 27d | >75%, Perifollicular |
| 5 | NSV | 45/Female | Left trunk | NB-UVB for 10 weeks | 35d | >75%, Marginal |
| 6 | NSV | 30/Male | Left shin | NB-UVB for 10 weeks | 30d | >75%, Combined |
| 7 | NSV | 18/Male | Left trunk | NB-UVB for 12 weeks | 24d | >75%, Combined |
| 8 | / | 28/Male | Left forearm | / | / | / |
| 9 | / | 30/Male | Right forearm | / | / | / |
| 10 | / | 29/Female | Left forearm | / | / | / |

NSV, non-segmental vitiligo;
